# A case report of bilateral temporomandibular joint ankylosis combined with dysplasia in cat

**DOI:** 10.5455/javar.2025.l899

**Published:** 2025-04-16

**Authors:** Yujie Ma, Xiaolin Xu, Lu Yi, Temesgen Roro Duresa

**Affiliations:** 1 Hunan Biological and Electromechanical Polytechnic, Changsha, China; 2 IVC Shenzhen Animal Hospital, Guangdong, China; 3 Holeta Polytechnic College, Holeta Town, Ethiopia

**Keywords:** Ankylosis, cat, CT, temporomandibular joint

## Abstract

**Objective:**

To describe the clinical presentation, diagnostic imaging findings, and management of a rare case of bilateral temporomandibular joint (TMJ) ankylosis combined with dysplasia in a cat. Materials and Methods: A 4-month-old Bombay male cat, normally immunized and dewormed. Radiographic studies: Details of imaging techniques used to diagnose TMJ ankylosis and dysplasia: computed tomography (CT) scans were used to visualize the joint structures in greater detail using CT.

**Results:**

Out of the 21 blood biochemical abnormalities found in the laboratory test, creatinine, albumin, albumin/globulin, and total cholesterol all showed a significant decrease. The levels of phosphorus and α-amylase were marginally elevated. A three-dimensional CT scan revealed a malformed fusion of the right TMJ zygomatic arch and bilateral mandibular coronal process. Both TMJ dysplasia and ankylosis were discovered by the biochemical and physical 3-dimensional CT scans.

**Conclusion:**

The findings underscore the importance of a thorough clinical examination and imaging studies to assess the extent of the ankylosis and any associated dysplastic changes. This case emphasizes the need for increased awareness of TMJ disorders in felines and encourages further research into effective treatment protocols.

## Introduction

A common clinical symptom of temporomandibular joint (TMJ) ankylosis is difficulty or inability to open the mouth. The disease is surgical in nature, and the normal mandibular joint structure is replaced by bone or fiber masses [1]. TMJ ankylosis in dogs and cats results in decreased mandibular mobility, especially obstruction of mouth opening and anterior and lateral movement. Fractures, dislocations, dysplasia, osteoporosis, arthritis, and other conditions frequently cause it as a secondary condition [2]. TMJ ankylosis can be divided into true and false; true ankylosis causes adhesion of bone fibers on the surface of the joint, which limits the range of motion of the joint and causes the joint to lose the ability to move. Non-traumatic TMJ ankylosis in cats occurs due to chronic inflammation, fibrous tissue proliferation, infection, degenerative changes, or immune-mediated diseases, leading to the fusion of the joint components and loss of mobility. Pseudo-rigidity is caused directly by the disease and has nothing to do with the joint itself [3]. Ankylosis of the TMJ may be intra-articular, extra-articular, or both [1]. TMJ stiffness can be caused by a variety of factors, including trauma, development, inflammation, and local tumors [4]. It has been reported that the incidence of TMJ ankylosis caused by trauma is higher in dogs and cats. Cats frequently sustain facial fractures and traumatic dislocations of the mandibular joint as a result of car accidents and falls [2,5].

The underlying causes of bilateral TMJ ankylosis and how they interact with dysplastic changes in the joint are not well understood. There may be genetic, congenital malformation, developmental, or traumatic factors involved [1]. Information about the clinical symptoms and indicators that define this condition in cats is lacking. Diagnostic criteria and imaging techniques specific to TMJ ankylosis and dysplasia in cats are not well established. There is a need for standardized protocols to enhance diagnostic accuracy. Bilateral TMJ ankylosis combined with dysplasia in cats is an exceptionally rare condition, with limited documented cases in the veterinary literature. This case provides unique insights into the clinical presentation, diagnostic challenges, and potential treatment approaches for this complex condition. Therefore, by filling in these knowledge gaps through focused case studies, cats with bilateral TMJ ankylosis and dysplasia may benefit from better diagnosis, care, and results. By highlighting the unique aspects of this case, we hope to contribute to the understanding of TMJ disorders in cats and provide a reference for veterinarians encountering similar cases in the future.

## Materials and Methods

### Ethical approval

The case described in this report was handled as part of the regular clinical caseload at the IVC Shenzhen Animal Hospital; as a result, neither institutional animal care and use committee nor ethical governing body approval was necessary. Owner consent was obtained for patient care in all aspects.

### Study design

This study employed a retrospective or prospective observational design, depending on the availability of cases. The study included clinical evaluations, imaging studies, and histopathological examinations.

## Case Presentation

### Basic information

The cat, a 4-month-old Bombay male, was regularly immunized and dewormed. The owner kept the cat for about 2 months. The cat could not open its mouth and eat solid food. So far, the cat only drank goat’s milk and had a strong appetite.

### Clinical examination

The mental state is normal; the body temperature is 38.4°C, the body weight is 0.8 kg, the heart rate is 180 beats/min, the breath is 40 breaths/min, and the mucosal color is pink. He has closed his mouth and is unable to open it. He is thin and malnourished.

### Diagnosis and treatment

**Fig. 1.**
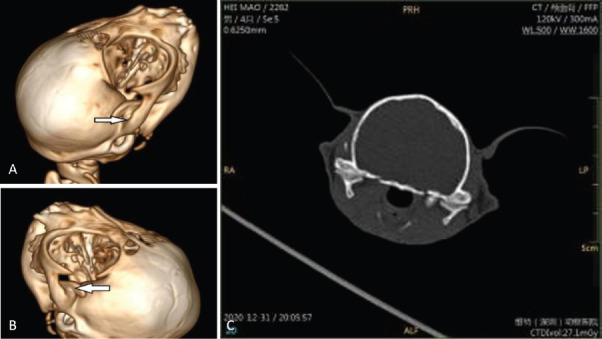
Figure 1. (A) 3D CT reconstruction, image on the right, arrows showing the malformed fusion of the right mandibular coronal process and zygomatic arch. (B) 3D CT reconstruction, left image, arrows show the malformed fusion of the left mandibular coronal process and zygomatic arch. (C) CT 2D sectional view.

Routine blood tests: Routine blood tests yielded no significant abnormalities. Table 1 shows the results of the blood biochemical test; six of them show abnormalities. Creatinine (CREA), phosphorus (P), albumin (ALB), and albumin/globulin (A/G) were decreased insignificantly. α-amylase (α-AMYα) and P were slightly increased. The computed tomography (CT) scan was performed under the irradiation condition of 300 mA and 120 kV. The results showed a malformed fusion of the bilateral mandibular coronal process and zygomatic arch. The results of the three-dimensional CT scan are shown in Figure 1A and B, and the results of the two-dimensional CT scan are displayed in Figure 1C.

**Table 1.** Results of blood test biochemical.

Test item	Results	Reference ranges
Glucose (Glu)	4.86	4.11–7.95 mmol/l
CREA	35.0↓	60.0–225.0 μmol/l
Urea nitrogen (UREA)	7.27	5.0–12.0 mmol/l
P	2.10↑	0.81–1.87 mmol/l
Calcium (Ca)	2.16	2.0–2.50 mmol/l
Total protein (TP)	64.9	52.0–95.0 gm/l
ALB	21.8↓	22.0–39.0 gm/l
Globin (GLOB)	43.1	25.0–45.0 gm/l
A/G	0.51↓	0.8–1.5
Alanine aminotransferase (ALT)	63.6	10.0–86.0 U/l
Alkaline phosphatase (ALP)	118.4	10.0–150.0 U/l
Aspartate aminotransferase (AST)	22.5	1.0–54.0 U/l
Glutamyl transpeptidase (GGTγ)	0.7	0.0–7.0 U/l
Total bile acid (TBA)	4.9	0.0–10.0 μmol/l
Total Bilirubine (TBIL)	0	0.0–15.0 μmol/l
Total CHOL	2.08↓	2.84–8.26 mmol/l
Triglyceride (TG)	0.89	0.09–1.6 mmol/l
α-AMYα	1,857.9↑	500–1800 U/l
Lipase (LIP)	0	0.0–258.0 U/l
Creatine kinase (CK)	218.1	26.0–450.0 U/l
Lactate dehydrogenase (LDH)	146.6	71.0–406.0 U/l

Diagnosis: according to the history, clinical examination, and imaging examination, it was confirmed that the cat had bilateral TMJ ankylosis combined with the malformed fusion of the bilateral coronal process and zygomatic arch.

### Treatment

**Fig. 2.**
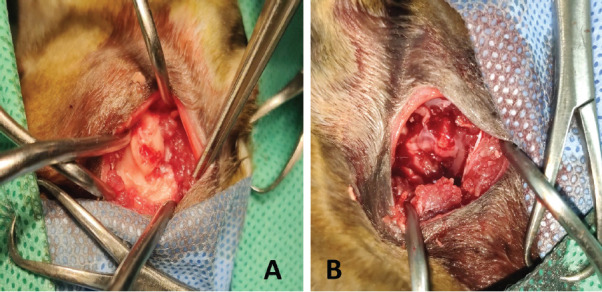
Figure 2. (A) Revealing the joint deformity fusion. (B) Removing the deformity fusion site.

**Fig. 3.**
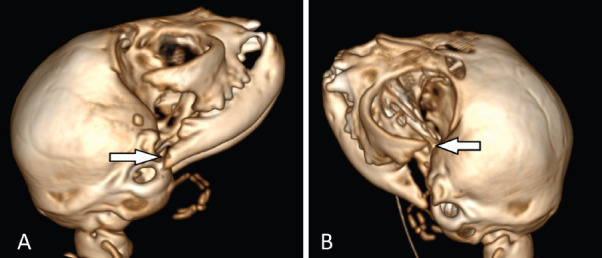
Figure 3. (A) Right postoperative view. Arrows show the removal of the posterior part of the right zygomatic arch, mandibular coronoid process, and its malformed fusion. (B) Left postoperative view. Arrows show the removal of the posterior part of the left zygomatic arch, the mandibular coronal process, and its malformed fusion.

The surgical treatment plan of bilateral TMJ space plasty was adopted. Anesthesia procedure antibiotics were routinely given, intravenous access was opened, butorphanol 0.2 mg/kg and propofol 3 mg/kg were injected intravenously, tracheotomy was performed on the supine, endotracheal intubation was inserted, and the respiratory anesthesia machine was connected for respiratory anesthesia; isoflurane concentration was maintained at 3% during anesthesia. The surgical site was aseptically prepared, and the animal was positioned in the right lateral recumbency. A curved skin incision approximately 4 cm in length was made along the ventrolateral aspect of the right zygomatic arch. The tissues and muscles were carefully dissected to identify and avoid the temporomandibular branch of the maxillary artery, transverse facial artery, and masseteric artery, as well as the medial pterygoid nerve, lingual nerve, and inferior alveolar nerve. The zygomatic arch and the coronoid process of the mandible were exposed, as shown in Figure 1C. Using bone rongeurs, the posterior portion of the zygomatic arch, the coronoid process of the mandible, and their malformed fusion were resected and completely removed, as illustrated in Figure 2. The surgical site was disinfected, and the incision was routinely closed. The same procedure was performed on the left-side TMJ. Postoperative examination revealed improved mobility of the jaw. A postoperative CT scan was conducted to assess the results. The postoperative CT scan was conducted to assess the results, as shown in Figure 3A and B.

Skin sutures were removed 7 days after surgery. After routine anti-inflammatory combined with analgesic treatment and a liquid diet for 2 days, the cat gradually began to eat normal cat food. One month after the operation, the weight of the cat had recovered to 1.8 kg. Six months after the operation, the cat was back in normal body condition. The patient showed a distinct improvement in both articular functionality and clinical signs.

## Discussion

Clinical signs of TMJ disease include pain during jaw movement, misaligned occlusion, and excessive salivation [6]. TMJ ankylosis is often secondary to trauma, tumor, and inflammation [3]. TMJ ankylosis can lead to malocclusion and craniofacial deformity in juveniles [1]. The canine TMJ consists of the condyle, fossa of the temporal bone, joint plate, and loose joint capsule, reinforced by lateral ligaments. The TMJ controls mouth opening, closing, and sliding motion. The joint plate in a cat has only a thin layer of fiberboard. A cat’s teeth can only move in one plane, not in a grinding motion [2]. The cat had no obvious history of trauma in this case, and the TMJ ankylosis was due to the malformed fusion of the posterior part of the zygomatic arch and the coronoid process of the mandible. This case is an unusual combination of bilateral TMJ ankylosis and dysplasia, which has not been extensively reported in feline medicine.

Additionally, the diagnostic imaging findings, surgical approach, and long-term outcomes provide new information that could contribute to the understanding and management of similar cases in the future.

### Diagnostic methods

Imaging is important for diagnosing the type and degree of structural deformation of TMJ ankylosis. Hyperplasia of bone in the TMJ can be observed [7] in the lateral, dorsoventral, and oblique positions on conventional X-rays. However, the TMJ is difficult to evaluate with conventional radiology due to structural and image distortions. CT imaging is an important means to evaluate TMJ stiffness and is the preferred method [4] for skeletal imaging of TMJ. A CT spiral scan can reconstruct a 3-dimensional model of the joint, and its images can provide precise and detailed information about the TMJ, including the type and degree of variation of the disease, and aid in the planning of surgery. In this case, we use the CT for detailed visualization of the structure. The 3D reconstruction of the TMJ provides a comprehensive view of the joint’s anatomy and abnormalities. For further treatment, these help the surgeon to better plan and carry out the surgery. Furthermore, integrating imaging findings with clinical signs such as restricted jaw movement and pain is crucial for accurate diagnosis.

In the results of the blood biochemistry test, four items results are lower than the reference range. CREA is a product of muscle metabolism and is produced primarily by muscle tissue. Urea nitrogen is the end product of protein metabolism and mainly comes from protein breakdown in the diet. Cholesterol (CHOL) is an important indicator of lipid metabolism, and its level is influenced by diet and liver function. ALB is the main protein synthesized by the liver, which reflects the nutritional status and protein reserve of the body. Due to the TMJ ankylosis combined with dysplasia in the cat, it can’t eat solid food for 2 months; only taking in milk cannot provide sufficient nutrition for it. Therefore, the malnourished young cats have inadequate protein, fat, and energy intake; decreased muscle mass; and low CREA, urea nitrogen, total CHOL, serum ALB, and albumin-to-globulin ratio.

As to slightly increased P, it may be related to increased cellular catabolism that releases intracellular P into the blood, leading to elevated blood P levels. The body may maintain an electrolyte balance in the blood by breaking down minerals in the bones, such as calcium and P. This increased bone catabolism leads to increased blood P levels. Besides, malnutrition may affect vitamin D metabolism, leading to increased intestinal absorption of P or decreased renal excretion of P. In this case, if a more detailed diagnosis or treatment is needed, other laboratory tests can be considered. The main role of α-AMYα is to break down carbohydrates, and in malnourished animals, amylase levels are usually not elevated but may be reduced. However, the α-AMYα in this cat is increased; further tests are recommended to determine the underlying cause.

### Treatment

In human medicine, there are three basic surgical methods [8] for the surgical treatment of TMJ ankylosis: 1) interjoint replacement (removal of bone mass combined with biological or non-biological materials); 2) joint reconstruction (removal of the bone mass and reconstruction of the structure with bone grafts or joint prostheses); and 3) joint space plasty (osteotomy) without the addition of other substances. Joint space arthroplasty is recommended for the surgical treatment of TMJ ankylosis in dogs and cats [9]. A number of articles have shown that joint space arthroplasty without joint replacement can successfully treat TMJ ankylosis [3]. The placement of interventional materials between the TMJ can isolate the broken ends, maintain the joint space, and prevent the recurrence of stiffness [10].

The temporalis muscle is the strongest in the heads of carnivores. The anatomical features of the temporalis muscle allow it to turn inward without affecting blood flow, thus ensuring the viability of the transplanted muscle flap. Temporal muscle fascial muscle flap is an effective method to repair various defects in the maxillofacial region [4]. Facial nerve palsy and joint ankylosis are common complications after this surgery. Overstretching of the periarticular tissue may lead to facial nerve palsy, inadequate physical therapy, and incomplete clearance of hyperplastic tissue in the joint space may lead to joint ankylosis [11]. Young dogs and cats have a relatively strong propensity for healing and callus formation, so surgical healing and interjoint re-adhesion due to extensive callus growth need to be considered [12].

Due to the severe atrophy of the temporal muscle, the transplantation of the muscle flap was not performed in this case. We performed the joint space plasty without the addition of other substances. Arthroplasty can adjust the joint space, preserve the patient’s natural joint structure, avoid artificial joint replacement, and reduce the risk of foreign body implantation. Less trauma means faster recovery and lower risk of post-op infections, suitable for patients in less physical condition. Arthroplasty costs less and reduces patient financial stress. Arthroplasty has significant advantages in the treatment of TMJ deformity, including the preservation of natural structure, less trauma, good functional recovery, light economic burden, and strong reversibility. The significance of the operation is to improve joint function, delay degeneration, improve quality of life, realize individualized treatment, and reduce complications. This cat gradually began to eat normal cat food; it only took 9 days after surgery, and it recovered to normal body condition soon. It showed distinct improvement in both the articular functionality and clinical signs. These advantages and the significance of arthroplasty make it an important choice for the treatment of TMJ ankylosis or deformity.

### Limitations

As this is a single case report, the findings and conclusions may not be generalizable to all cats with similar conditions. The rarity of the condition limits the ability to draw broad conclusions or establish standardized treatment protocols. Since there is no histopathological analysis of the affected joint, the results could limit the understanding of the underlying pathological processes contributing to the ankylosis and dysplasia. The follow-up period was short or incomplete; this could limit the ability to assess long-term outcomes, such as recurrence of ankylosis, functional recovery, or potential complications.

## Conclusion

Arthroplasty (bone resection) is a better method for treating TMJ ankylosis in young cats. Combining 3D CT imaging to assess the condition of the TMJ can provide precise information about it and thereby improve the surgical outcome. Young animals heal quickly and can form bone calluses well, so it is important to think about how the surgery will heal and the large bone callus growth that might lead to the joints sticking together again.

## List of abbreviations

A/G, Albumin/Globulin

ALB, Albumin

CHOL, Cholesterol

CREA, Creatinine

CT, Computed Tomography

P, Phosphorus

TMJ, temporomandibular joint

α-AMY, α-Amylase.
